# Predictors of lower-limb arterial occlusion pressure across commonly used cuff widths

**DOI:** 10.3389/fphys.2025.1658744

**Published:** 2025-10-10

**Authors:** Isaac J. Wedig, Isaac M. Lennox, Erich J. Petushek, John J. Durocher, John McDaniel, Steven J. Elmer

**Affiliations:** ^1^ School of Health and Human Performance, Northern Michigan University, Marquette, MI, United States; ^2^ Department of Kinesiology and Integrative Physiology, Michigan Technological University, Houghton, MI, United States; ^3^ Health Research Institute, Michigan Technological University, Houghton, MI, United States; ^4^ Department of Psychology and Human Factors, Michigan Technological University, Houghton, MI, United States; ^5^ Department of Biological Sciences and Integrative Physiology and Health Sciences Center, Purdue University Northwest, Hammond, IN, United States; ^6^ Department of Exercise Physiology, Kent State University, Kent, OH, United States; ^7^ Doctor of Physical Therapy Program, St. Catherine University, Saint Paul, MN, United States

**Keywords:** blood flow restriction, vascular occlusion, kaatsu, systolic blood pressure, limb occlussion pressure

## Abstract

We compared predictors of lower-limb arterial occlusion pressure (AOP) across commonly used blood flow restriction (BFR) cuff widths (11, 13, 18 cm) and developed prediction equations to estimate AOP for each cuff. Participants (n = 116) underwent measurements of thigh circumference (TC), systolic (SBP) and diastolic (DBP) blood pressure, and AOP was assessed using Doppler ultrasound in a seated position. Multiple linear regression models with commonality analysis and mixed-effects models were used to identify and compare predictors of AOP between each cuff. LASSO regression with bootstrap resampling was used to develop and internally validate prediction equations. TC, SBP, DBP, age, and sex explained 60%–70% of total variance in AOP, with greater predictive power in narrower cuffs. As cuff width increased, TC uniquely accounted for less (36%, 26%, 11% for 11, 13, 18 cm, respectively) and SBP uniquely accounted for more (2%, 6%, 12% for 11, 13, 18 cm, respectively) variance. A cuff width × TC interaction indicated that limb size had greater influence on AOP with narrower cuffs. In contrast, the relationship between SBP and AOP remained stable across cuff widths. Prediction equations demonstrated good predictability and calibration, with limits of agreement ranging from ±18.4 to ±28.6 mmHg and statistical equivalence between predicted and measured AOP. Internal validation showed minimal overfitting. These findings highlight the importance of accounting for cuff width in BFR pressure prescription, with narrower cuffs requiring consideration primarily of TC, and wider cuffs requiring consideration of both TC and SBP. These cuff-specific equations may offer a practical alternative to direct AOP measurement.

## Introduction

For the implementation of exercise with blood flow restriction (BFR), it is recommended that cuff pressures be selected based on arterial occlusion pressure (AOP) (Patterson et al., 2019) which is the pressure required to occlude arterial blood flow to a limb. Selecting pressures relative to AOP ensures a standardized level of arterial restriction during exercise, typically within the 40%–80% AOP range, which is considered both safe and effective. Clinicians, coaches, and athletes, however, may not have access to equipment needed for directly assessing AOP (i.e., Doppler ultrasound, handheld Doppler, cuffs with pressure sensors). To develop alternative methods for establishing cuff pressures, several authors (Cirilo-Sousa et al., 2019; Jessee et al., 2016; Loenneke et al., 2015; Loenneke et al., 2012; Sieljacks et al., 2018; Wedig et al., 2024) have investigated anthropometric, hemodynamic, and sociodemographic variables as predictors of AOP. Reports (Cirilo-Sousa et al., 2019; de Queiros et al., 2024; Loenneke et al., 2015; Loenneke et al., 2012; Sieljacks et al., 2018; Wedig et al., 2024) focusing on the lower-limbs indicate that measures of thigh circumference (TC) and various measures of blood pressure, including brachial systolic (SBP) and diastolic blood pressure (DBP), serve as the strongest predictors of AOP, collectively explaining ∼40–70% of the variance between individuals.

It is important to point out that a wide range of cuff widths are used for implementing exercise with BFR (i.e., 3–18 cm) (Patterson et al., 2019) and predictors of AOP may vary between different cuff widths. For example, de Queiros et al. (2024) recently reported that TC was the main predictor of lower-limb AOP when utilizing an 11 cm cuff while SBP was the main predictor when utilizing an 18 cm cuff. In related work, Crenshaw et al. (1988) demonstrated that lower-limb AOP is more dependent upon TC in narrower cuffs when compared to wider cuffs. These authors reported that Pearson correlations between TC and AOP for 4.5, 8, 12, and 18 cm cuffs were 0.89, 0.82, 0.77, and 0.44, respectively. There was no relationship between SBP and AOP in any of the cuff widths studied. Conversely, Loenneke et al. (2012) reported that AOP was more dependent on TC with a 13.5 cm (r = 0.71) versus a 5 cm (r = 0.40) cuff. These authors also reported no relationship between brachial SBP and AOP. Alternatively, data from separate reports describing predictors of lower-limb AOP in 13.5 cm (Loenneke et al., 2015) and 18 cm (Wedig et al., 2024) cuffs suggest that brachial SBP is the strongest predictor of AOP when utilizing a wider cuff. Based on these varied findings, the extent to which predictors of AOP vary across cuff widths remains unclear. Identifying the strongest predictors of AOP across commonly used cuff widths may support the development of alternative methods, such as prediction equations, for selecting BFR pressures without direct measurement.

Several multivariate prediction equations utilizing combinations of predictors such as TC, SBP, DBP, age, and sex have been developed for estimating lower-limb AOP in cuff widths ranging from 5–18 cm (Cirilo-Sousa et al., 2019; Jessee et al., 2016; Loenneke et al., 2015; Loenneke et al., 2012; Sieljacks et al., 2018). These prediction equations provide a practical way to implement exercise with BFR, however, methodological quality and insufficient reporting of performance measures pose limitations to their use. Additionally, most equations have been developed to predict AOP in a supine position and thus may not provide accurate estimates of AOP in exercising body positions (de Queiros et al., 2024; Sieljacks et al., 2018). Recently, we demonstrated good predictability for an equation to estimate AOP for an 18 cm cuff in a seated position (Wedig et al., 2024). Accordingly, similar methods for prediction equation development and performance reporting are needed for additional cuff widths in the seated position.

The purpose of this study was to 1) compare predictors of lower-limb AOP across a range of cuff widths commonly used for implementing exercise with BFR and 2) develop and validate equations to predict AOP when applying these cuffs in a seated position. For Part 1 of this study, we compared the relationship between TC and measures of blood pressure (SBP and DBP) with AOP taken using 11, 13, and 18 cm cuffs. Based on previous reports (Crenshaw et al., 1988; de Queiros et al., 2024; Wedig et al., 2024), we hypothesized that TC would be a stronger predictor of AOP when using narrower cuffs whereas blood pressure would be a stronger predictor when using wider cuffs. For Part 2 of this study, we developed a prediction equation for each cuff width and internally validated the resulting models to assess the stability of their performance within our data. Ultimately, this work aimed to increase accessibility to safe and effective BFR exercise by providing practical methods to estimate AOP and standardize cuff pressure selection.

## Methods

### Participants

One hundred sixteen healthy adults between the ages of 18 and 39 years were recruited to participate in this study (Table 1). Data collection occurred during the COVID-19 pandemic and in-between case surges. In an effort to include a larger number of females, menstrual cycle was not controlled for. Participants were excluded from the study if they had a body mass index (BMI) >35 kg/m^2^, SBP >140 mmHg, DBP >90 mmHg, used nicotine products, had any cardiometabolic, dermatological, or neurological disorders, had a recent lower-limb injury or surgery, or had any implanted devices. Participants were informed of the purpose of the study, the risks involved, and provided informed written consent. This study was reviewed and approved by the Institutional Review Board at Michigan Technological University.

**TABLE 1:** Participant characteristics (Male: n = 68, Female: n = 48).

Variable	Mean ± SD	Minimum	Maximum
Age (years)	23 ± 5	18	39
Height (m)	1.7 ± 0.1	1.5	2.0
Body mass (kg)	74.8 ± 12.6	48.0	114.0
BMI (kg/m^2^)	24.6 ± 3.3	17.9	34.4
TC (cm)	60.7 ± 5.0	50.0	78.8
SBP (mmHg)	122 ± 9	95	138
DBP (mmHg)	74 ± 7	57	89
11 cm AOP (mmHg)	185 ± 26	136	277
13 cm AOP (mmHg)	168 ± 19	131	228
18 cm AOP (mmHg)	150 ± 14	111	190

AOP , arterial occlusion pressure; DBP, diastolic blood pressure; SBP, systolic blood pressure; TC , thigh circumference.

### Study design and overview

**FIGURE 1:**
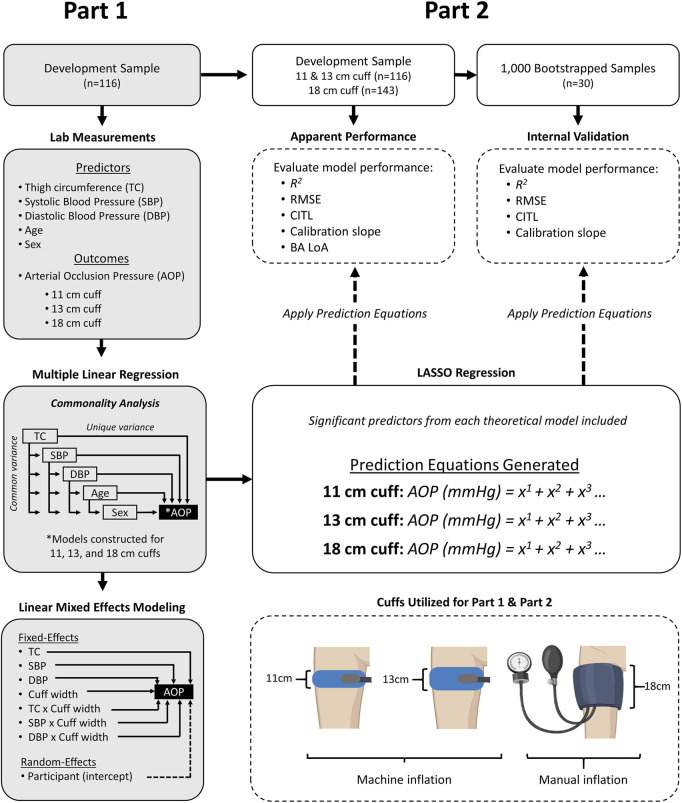
Overview of study design. Part 1- methods for identifying and comparing predictors of AOP in 11, 13, and 18 cm cuffs. Part 2 - methods for developing and internally validating prediction equations to estimate AOP in 11, 13, and 18 cm cuffs. AOP = Arterial Occlusion Pressure, BA LoA = Bland-Altman Limits of Agreement, CITL = Calibration-in-the Large, DBP = Diastolic Blood Pressure, SBP = Systolic Blood Pressure, RMSE = Root mean squared error.

In this investigation, we used a cross-sectional study design as participants visited the laboratory for one experimental testing session. Participants were asked to avoid caffeine consumption and strenuous exercise for 8 h prior to the visit and to refrain from eating at least 2 h prior. Upon arrival, height and body mass were measured. Next, TC was measured, followed by SBP and DBP. Finally, lower-limb AOP was determined in the seated position using an 11, 13, and 18 cm cuff in a randomized order. For Part 1 of this study, multiple linear regression models were constructed to predict AOP in the lower limb for each cuff using the predictor variables TC, SBP, DBP, age, and sex. Commonality analysis was used to compare the unique and shared percentage of variance in AOP that was associated with each predictor variable across all three cuff models. A linear mixed effect model was then used to further investigate the relationship between predictor variables and AOP across the different cuff widths. For Part 2 of this study, we used methods recently described by our laboratory (Wedig et al., 2024) to develop and internally validate prediction equations to estimate AOP for 11, 13, and 18 cm cuffs. Note that for the 18 cm cuff only, data from an additional 27 participants from our previous work (Wedig et al., 2024) were included to develop and validate this prediction equation. An overview of the study design is presented in Figure 1.

### Thigh circumference

Thigh circumference was obtained on the right leg of each participant at 33% of the distance from the inguinal crease to the proximal patella using a standard tape measure. This measurement location was selected to represent the site at which the cuffs were placed during AOP measurement. Measures were taken in duplicate to the nearest millimeter, and the average value was used for analysis. Based on preliminary data, reliability of circumference measures (ICC = 0.97) were excellent, which is consistent with previously reported data (Bakar et al., 2017; Wedig et al., 2024).

### Blood pressure

After resting quietly in a seated position for 10 min, brachial SBP and DBP were obtained using an appropriately sized automatic blood pressure cuff (Welch-Allyn, Model 4200B-E1, Skaneateles Falls, NY, United States). All blood pressure measures were obtained with the participant in a seated position. A minimum of two measurements were taken with a 1 min rest between measures. If SBP or DBP varied by more than 5 mmHg, measurements were repeated until values were within 5 mmHg of each other. The two sequential values within 5 mmHg were averaged and used for analysis.

### Arterial occlusion pressure

In a randomized order, measures of lower-limb AOP were assessed with an 11 cm (SC10D, 11 × 85 cm, Hokanson, Bellevue, WA, United States), 13 cm (SC12D, 13 × 85 cm, Hokanson, Bellevue, WA, United States), and 18 cm (Thigh Size Aneroid Sphygmomanometer, Elite Medical Instruments, Fullerton, CA, United States) pneumatic cuff. All cuffs were constructed of a rigid nylon material with a straight segmental shape. The 11 and 13 cm cuffs were inflated using a rapid cuff inflation system (E20 Rapid Cuff Inflation System, Hokanson, Bellevue, WA, United States) while the 18 cm cuff was manually inflated by hand. An 18 cm manually inflated cuff was used to maintain consistency in cuff shape, as the only 18 cm cuff available from the same manufacturer as the 11 and 13 cm cuffs has a contoured design. Measures of lower-limb AOP were obtained by first placing the cuff on the proximal portion of the thigh with the center of the bladder positioned at 33% of the distance from the inguinal crease to the proximal patella. Next, the participant was placed in a seated position and the pulse was detected at the posterior tibial artery using Doppler ultrasound (GE Logiq e BT12, GE Healthcare, Chicago, IL, United States). The cuff was first inflated to 75 mmHg and the pressure was slowly increased at a rate of 2–3 mmHg/s until blood velocity in the posterior tibial artery reached zero based on the absence of the Doppler spectrum. Once an initial blood velocity of zero was reached, the cuff pressure was maintained for approximately 5–10 s to confirm full occlusion. If blood flow resumed during this period, inflation was continued until blood velocity again reached zero. The minimum pressure required fully to eliminate Doppler spectrum was recorded as the AOP. A minimum of two measures were obtained for each cuff with a 2 min break between measures. If values varied by more than 5 mmHg, measurements were repeated until values were within 5 mmHg. The two consecutive values within 5 mmHg were averaged and used for analysis. Participants were given a 2 min break between measures of AOP with each cuff. Based on preliminary data, measurement of AOP using this method was reliable (SEM = 2.01 mmHg, ICC = 0.95) and is consistent with previous reliability data reported from our laboratory (Kilgas et al., 2019a; Kilgas et al., 2019b; Kilgas et al., 2022; Wedig et al., 2024).

### Statistical analysis

#### Part 1- Predictors of AOP

Multiple linear regression models were constructed to predict lower-limb AOP for each of the three cuffs using the predictor variables of TC, SBP, DBP, age, and sex. These variables were selected as they have been previously identified as predictors of AOP which collectively explain 40%–70% of the total variance (Cirilo-Sousa et al., 2019; de Queiros et al., 2024; Loenneke et al., 2015; Loenneke et al., 2012; Wedig et al., 2024), corresponding to a large effect size (Cohen’s f^2^ > 0.35) (Cohen, 2013). Given a power of 0.8 (ß = 0.20) and a two-tailed significance level (α) of 0.05, we determined that 91 participants would provide an adequate sample to detect a medium effect size (f^2^) of 0.15 with five predictor variables. One theoretical model including all predictors was constructed for each cuff. Model fit was evaluated using coefficient of determination (*R* ^*2*^), adjusted coefficient of determination (Adj *R* ^*2*^), standard error of the estimate (SEE), mean squared error (MSE), and an F-test to determine whether the full set of predictors explained a significant proportion of variance in AOP. Individual predictors were evaluated using their respective t-tests and p-values, with an alpha level set at 0.05. Standardized beta coefficients (β) were reported to compare the relative contributions of each predictor. Assumptions of linear models were checked with a visual inspection of normality and residual plots. Multi-collinearity between predictor variables was assessed using variance inflation factor (VIF) and Pearson’s correlations. Multi-collinearity was defined as a VIF ≥5 and/or Pearson’s correlations of 0.85 or greater. To assess the unique and shared variance explained by each predictor in the linear regression models, we conducted a commonality analysis (Nathans et al., 2012). The analysis was performed using the ‘yhat’ package (Nimon et al., 2009) available on R (R: A Language and Environment for Statistical Computing, 2020, R Foundation for Statistical Computing, Vienna, Austria). To further compare the influence of TC, SBP, and DBP on AOP across the different cuff widths we fit a linear mixed effects model. The model included AOP as the outcome variable with fixed effects of TC, SBP, DBP, cuff width, TC x cuff width, SBP x cuff width, and DBP x cuff width, and a random intercept for participants. Age and sex were not included as fixed effects given the limited sample size. The random intercept for participants accounts for some inter-individual variability potentially related to age and sex. Additionally, while age and sex have previously been identified as predictors of AOP, findings are inconsistent, and these variables typically explain minimal variance when considered alongside limb circumference and blood pressure. Given this, we focused our analysis on the more influential predictors. The mixed effects model was constructed using the ‘lme4’ package (Bates et al., 2015) available on R and alpha was set to 0.05.

#### Part 2- Developing and validating prediction equations

Data from all participants were used to develop equations to predict AOP for the 11, 13, and 18 cm cuffs. The minimum sample size for developing the prediction equation was determined using criteria described by Riley et al. (2019) for prediction models with continuous outcome variables. Based on preliminary data (*n* = 88) utilizing a model with five predictor variables, an adjusted *R* ^2^ of 0.40, and a mean AOP of 154 mmHg and standard deviation of 13 mmHg, a sample of 140 participants was determined to control model optimism (shrinkage < 10%) and provide estimates of the residual standard deviation and model intercept with a margin of error less than 20%.

We followed the transparent reporting of a multivariable model for individual prognosis or diagnosis (TRIPOD) guidance for development and reporting of multivariable prediction models (Moons et al., 2015). Least absolute shrinkage and selection operator (LASSO) regression was carried out for the 11, 13, and 18 cm cuffs and included each of the significant predictor variables that were identified in Part 1 as candidate predictors. The optimal penalization parameter (λ) was selected using automated 30-fold cross-validation to determine the λ that minimizes mean-squared error in the model. Variables with coefficients that were reduced to zero after regularization were removed from the model. Performance of the resulting models were evaluated in the development dataset by assessing model *R* ^*2*^ (the proportion of variance in AOP explained by the model), model root-mean squared error (RMSE, the average difference between the predicted and observed values), calibration slope (slope from a model regressing observed on predicted AOP values; ideal value is 1), and calibration-in-the large (CITL, the intercept term from a model regressing observed on predicted AOP values; ideal value is 0). The degree of agreement between observed and predicted AOP values obtained from the prediction equations was assessed using techniques described by Bland and Altman (1986). The 95% limits of agreement were determined by calculating two standard deviations of the mean difference between observed and predicted values and 95% confidence intervals (CI) were constructed around the limits of agreement (Giavarina, 2015). To statistically test the equivalence of mean observed and predicted AOP values we utilized a two one-sided t-test (TOST; 90% confidence interval, or 5% for each lower and upper limit) described by Lakens (2017). The equivalence region was selected as ± 10% of the mean AOP determined via Doppler ultrasound. This equivalence region was selected arbitrarily as a 10% error in AOP likely has little practical importance. The test was carried out using the TOST package available on R from the CRAN repository (Mara and Cribbie, 2012).

Finally, internal validation was completed for each model using bootstrap resampling methods in which 1,000 samples (n = 30) were randomly selected from the development dataset with replacement. The prediction equation developed from the full dataset was applied to each of the random samples and used to predict AOP. The variable selection process was not included in internal validation as this was performed via LASSO regression. The performance of the model across validation samples was assessed by evaluating the distributions of model *R* ^*2*^, RMSE, calibration slope, and CITL obtained from the set of random samples. All data analysis was completed using R.

## Results

### Part 1- predictors of AOP

**FIGURE 2:**
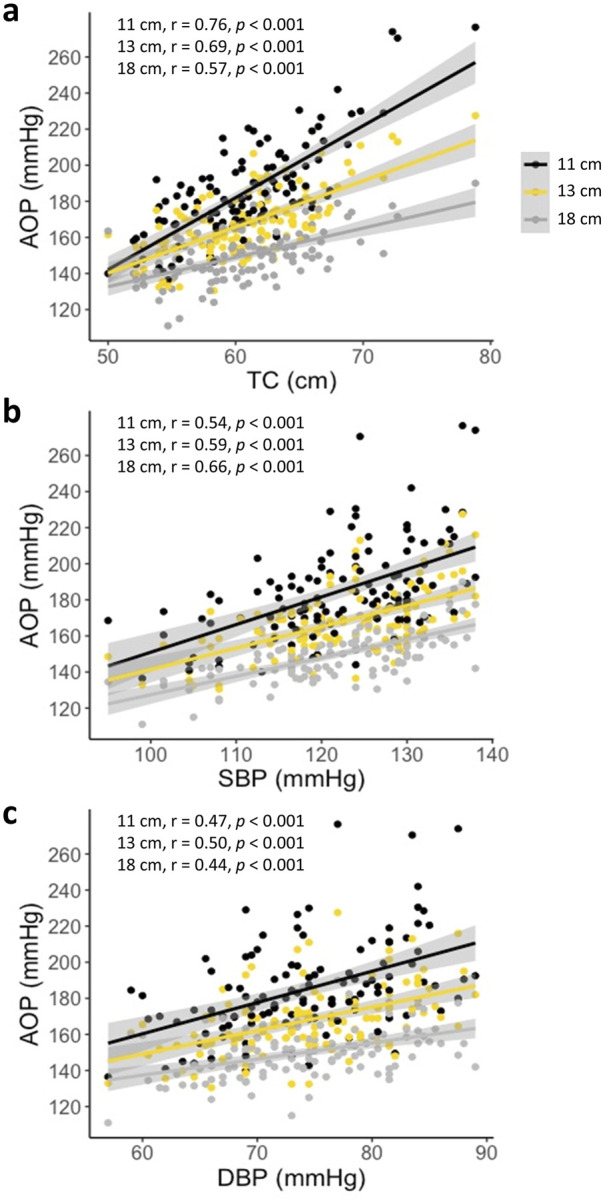
AOP versus predictor variables for the 11, 13, and 18 cm cuffs **(a)** AOP vs. TC **(b)** AOP vs. SBP **(c)** AOP vs. DBP. Significant effects of TC, SBP, DBP, cuff width, TC x cuff width, and DBP x cuff width (all *p* < 0.01). Pearson’s correlations (r) between the respective predictor variable and AOP are presented in the upper left corner of each plot.

One hundred and sixteen adults participated in Part 1 of this investigation. Participant characteristics are presented in Table 1. Results of the multiple linear regression analysis for the 11 cm cuff are presented in Table 2. The model explained 70% of the total variance in AOP, with TC (ß = 0.646, Part = 3.373), SBP (ß = 0.231, Part = 0.655), and DBP (ß = 0.165, Part = 0.610) constituting significant predictors. Results of the multiple linear regression analysis for the 13 cm cuff are presented in Table 3. The model explained 66% of the total variance in AOP, with TC (ß = 0.550, Part = 2.025), SBP (ß = 0.367, Part = 0.735), and sex (ß = −0.141, Part = −5.263) constituting significant predictors. Results of the multiple linear regression analysis for the 18 cm cuff are presented in Table 4. The model explained 61% of the total variance in AOP, with TC (ß = 0.363, Part = 1.031), SBP (ß = 0.536, Part = 0.827), and age (ß = 0.179, Part = 0.570) constituting significant predictors. Results of commonality analysis are shown in Table 5. In the 11 cm cuff, TC accounted for the largest portion of variance in AOP (58.3%), with a substantial unique contribution (36.1%). SBP contributed 29.2% to the total variance, though most of this was shared with other predictors (26.9%). In the 13 cm cuff, TC remained the strongest overall predictor (48.2% total; 26.2% unique) while SBP contributed 34.9% to the total variance with a unique contribution of 5.7%. For the 18 cm cuff, SBP accounted for the greatest variance in AOP (44.0%), with its unique contribution (12.2%) surpassing that of TC (11.4%). TC’s overall contribution decreased to 32.8%. The variables DBP, age, and sex explained minimal variance across cuff sizes, with most of their effects being shared with other predictors. The linear mixed effects model revealed significant main effects of TC (p < 0.001), SBP (p < 0.001), DBP (p < 0.01), cuff width (p < 0.001), TC x cuff width interaction (p < 0.001), and DBP x cuff width interaction (p < 0.01) (Figure 2a–c).

**TABLE 2:** Linear regression model for 11 cm cuff.

Predictor	Stand. ß	*p* value	Part
TC	0.646	< 0.001	3.373
SBP	0.231	< 0.01	0.655
DBP	0.165	< 0.05	0.610
Age	0.021	0.698	0.121
Sex	−0.060	0.346	−3.159

*R* ^*2*^	*Adj. R* ^2^	MSE	SEE	*p* value
0.695	0.681	208.50	14.83	< 0.001

DBP, diastolic blood pressure; SBP, systolic blood pressure; TC, thigh circumference.

**TABLE 3:** Linear regression model for the 13 cm cuff.

Predictor	Stand. ß	*p* value	Part
TC	0.550	< 0.001	2.025
SBP	0.367	< 0.001	0.735
DBP	0.128	0.108	0.334
Age	−0.018	0.752	−0.074
Sex	−0.141	< 0.05	−5.263

*R* ^*2*^	*Adj. R* ^2^	MSE	SEE	*p* value
0.660	0.645	115.40	11.03	< 0.001

DBP, diastolic blood pressure; SBP, systolic blood pressure; TC, thigh circumference.

**TABLE 4:** Linear regression model for 18 cm cuff.

Predictor	Stand. ß	*p* value	Part
TC	0.363	<0.001	1.031
SBP	0.536	<0.001	0.827
DBP	0.007	0.937	0.013
Age	0.179	<0.01	0.570
Sex	−0.053	0.460	−1.526

*R* ^*2*^	*Adj. R* ^2^	MSE	SEE	*p* value
0.606	0.588	79.47	9.16	<0.001

DBP, diastolic blood pressure; SBP, systolic blood pressure; TC, thigh circumference.

**TABLE 5:** Results of commonality analysis for regression models predicting AOP in 11, 13, and 18 cm cuffs.

Predictor variable	11 cm cuff			13 cm cuff			18 cm cuff		
	*Unique*	*Common*	*Total*	*Unique*	*Common*	*Total*	*Unique*	*Common*	*Total*
TC	0.361	0.222	0.583	0.262	0.221	0.482	0.114	0.214	0.328
SBP	0.023	0.269	0.292	0.057	0.292	0.349	0.122	0.318	0.440
DBP	0.013	0.207	0.220	0.008	0.243	0.251	0.000	0.198	0.198
Age	0.000	0.018	0.019	0.000	0.008	0.008	0.031	0.039	0.070
Sex	0.003	0.002	0.003	0.014	−0.014	0.000	0.002	0.025	0.027

DBP, diastolic blood pressure; SBP, systolic blood pressure; TC, thigh circumference.

### Part 2 - Developing and validating prediction equations

#### Prediction equation development

When performing LASSO regression to predict AOP for the 11 cm wide cuff the variables of TC, SBP, and DBP were entered as candidate predictors. The optimal value to use for λ was determined to be 0.844. Coefficients for TC, SBP, and DBP remained non-zero after regularization and thus these predictor variables were retained in the final model. The representative formula for the resulting equation when using the 11 cm cuff was:

$$\begin{array}{l} AOP\;\left(mmHg\right)=-124.750+3.269\;\left(Thigh\;Circumference\right)\\ \;+\;0.506\;\left(SBP\right)+0.6647\;\left(DBP\right) \end{array}$$

When performing LASSO regression to predict AOP for the 13 cm cuff the variables of TC, SBP, and sex were entered as candidate predictors. The optimal value to use for λ was determined to be 0.053. Coefficients for all candidate predictors remained non-zero after regularization and thus all predictor variables were utilized in the final model. The representative formula for the resulting equation when using the 13 cm cuff was:

$$\begin{array}{l} AOP\;\left(mmHg\right)=-55.636+2.017\;\left(Thigh\;Circumference\right)\\ \;+\;0.915\;\left(SBP\right)\;–\;6.960\;\left(Sex,2=male,1=female\right) \end{array}$$

For the 18 cm cuff, the variables of SBP, TC, and age were entered as candidate predictors in LASSO regression. The optimal value to use for λ was determined to be 0.042. The coefficients for each predictor variable remained non-zero after regularization and thus all predictor variables were retained in the final model. The representative formula for the resulting equation when using the 18 cm cuff was:

$$\begin{array}{l} AOP\;\left(mmHg\right)=-12.179+1.084\;\left(Thigh\;Circumference\right)\\ \;+\;0.720\;\left(SBP\right)+0.426\;\left(Age\right) \end{array}$$

#### Apparent performance in development data

**FIGURE 3:**
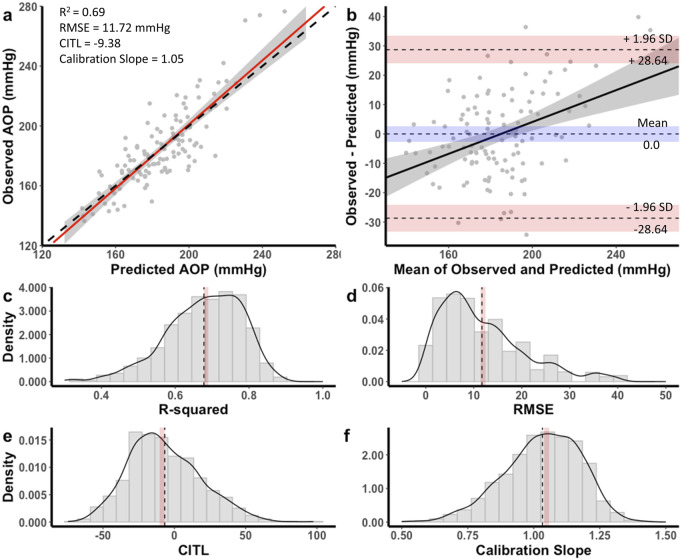
Performance of equation to predict AOP for the 11 cm cuff in the development dataset **(a,b)** and distribution of performance measures across internal validation samples **(c–f)**. **(a)** Calibration plot of observed versus predicted AOP values. Black dashed line represents the ideal trendline (b = 0, slope = 1). Red solid line represents the line of best ft between observed and predicted values and gray shaded region is the 95% CI. **(b)** Bland–Altman plot displaying the limits of agreement between observed and predicted AOP values. Black dashed lines represent the mean difference and upper and lower limits of agreement (95% interval). Shaded regions represent 95% CIs. Black solid line is a regression ft of the difference on the means and gray shaded region is the 95% CI. **(c)** Model *R* ^2^, **(d)** root mean square error (RMSE), **(e)** calibration-in-the large (CITL), **(f)** calibration slope. Note that black dashed lines **(c–f)** represent distribution means and red shaded regions represent 95% CIs.

The equation developed for the 11 cm cuff explained 69% of the total variance in AOP (*R* ^*2*^ *adj* = 0.68) with an RMSE of 11.72 mmHg. The CITL and calibration slope were −9.38 and 1.05, respectively (Figure 3a). A Bland-Altman plot displaying the limits of agreement between measured and predicted AOP values is presented in Figure 3b. The estimated mean difference between values was 0.0 mmHg, 95% CI [-0.29, 0.29]. The upper and lower 95% limits of agreement were 28.64 mmHg, 95% CI [24.0, 33.3] and −28.64 mmHg, 95% CI [-33.3, −24.0]. The model displayed proportional bias as the slope of the regression of the differences of observed and predicted values by the mean of values was different from 0 (0.25, 95% CI [0.14, 0.36], p < 0.001). Observed and predicted AOP values were equivalent, *t* (115) = −8.616, *p* < 0.001, given an equivalence region −11.69 to +11.69 mmHg. The 90% confidence interval for the mean difference between observed and predicted AOP values was −2.25 and +2.25 mmHg, which was well within the selected equivalence region.

**FIGURE 4:**
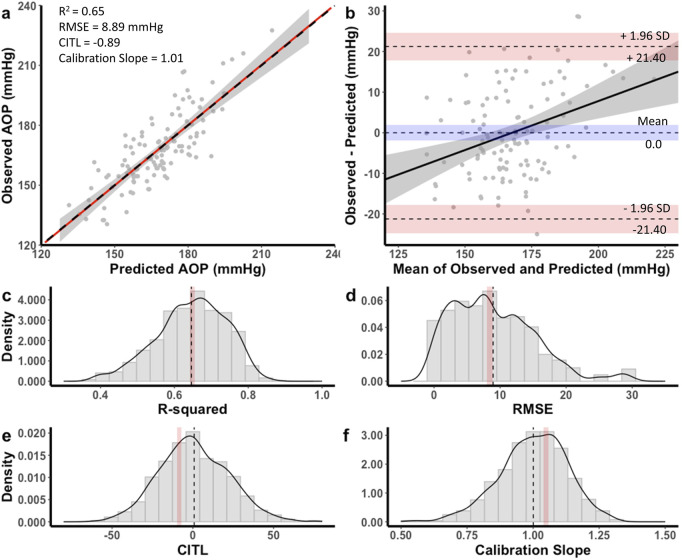
Performance of equation to predict AOP for the 13 cm cuff in the development dataset **(a,b)** and distribution of performance measures across internal validation samples **(c–f)**. **(a)** Calibration plot of observed versus predicted AOP values. Black dashed line represents the ideal trendline (b = 0, slope = 1). Red solid line represents the line of best ft between observed and predicted values and gray shaded region is the 95% CI. **(b)** Bland–Altman plot displaying the limits of agreement between observed and predicted AOP values. Black dashed lines represent the mean difference and upper and lower limits of agreement (95% interval). Shaded regions represent 95% CIs. Black solid line is a regression ft of the difference on the means and gray shaded region is the 95% CI. **(c)** Model *R* ^2^, **(d)** root mean square error (RMSE), **(e)** calibration-in-the large (CITL), **(f)** Calibration slope. Note that black dashed lines **(c–f)** represent distribution means and red shaded regions represent 95% CIs.

The equation developed for the 13 cm cuff explained 65% of the total variance in AOP (*R* ^*2*^ *adj* = 0.64) with an RMSE of 8.89 mmHg. The CITL and calibration slope were −0.89 and 1.01, respectfully (Figure 4a). A Bland-Altman plot displaying the limits of agreement between measured and predicted AOP values is presented in Figure 4b. The estimated mean difference between values was 0.0 mmHg, 95% CI [-0.21, 0.21]. The upper and lower 95% limits of agreement were 21.40 mmHg, 95% CI [17.9, 24.9] and −21.40 mmHg, 95% CI [-24.9, −17.9]. The model displayed proportional bias as the slope of the regression of the differences of observed and predicted values by the mean of values was different from 0 (0.24, 95% CI [0.12, 0.36], p < 0.001). Observed and predicted AOP values were equivalent, *t* (115) = −8.616, *p* < 0.001, given an equivalence region −8.74 to +8.74 mmHg. The 90% confidence interval for the mean difference between observed and predicted AOP values was −1.68 and +1.68 mmHg, which was well within the selected equivalence region.

**FIGURE 5:**
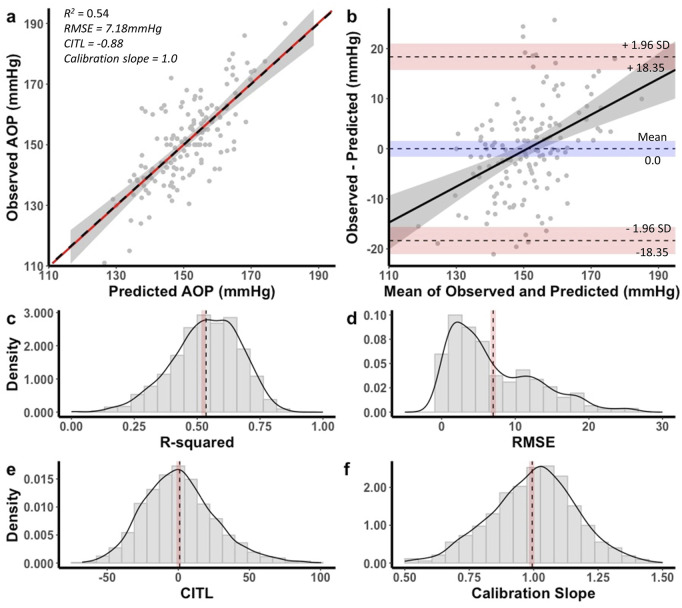
Performance of equation to predict AOP for the 18 cm cuff in the development dataset **(a,b)** and distribution of performance measures across internal validation samples **(c–f)**. **(a)** Calibration plot of observed versus predicted AOP values. Black dashed line represents the ideal trendline (b = 0, slope = 1). Red solid line represents the line of best ft between observed and predicted values and gray shaded region is the 95% CI. **(b)** Bland–Altman plot displaying the limits of agreement between observed and predicted AOP values. Black dashed lines represent the mean difference and upper and lower limits of agreement (95% interval). Shaded regions represent 95% CIs. Black solid line is a regression ft of the difference on the means and gray shaded region is the 95% CI. **(c)** Model *R* ^2^, **(d)** root mean square error (RMSE), **(e)** calibration-in-the large (CITL), **(f)** calibration slope. Note that black dashed lines **(c–f)** represent distribution means and red shaded regions represent 95% CIs. Figure reprinted with permission from Wedig et al. (2024).

The equation developed for the 18 cm cuff explained 54% of the total variance in AOP (*R* ^*2*^ *adj* = 0.53) with an RMSE of 7.18 mmHg. The CITL and calibration slope were −0.88 and 1.01, respectfully (Figure 5a). A Bland-Altman plot displaying the limits of agreement between measured and predicted AOP values is presented in Figure 5b. The estimated mean difference between values was 0.0 mmHg, 95% CI [-1.55, 1.55]. The upper and lower 95% limits of agreement were 18.35 mmHg, 95% CI [15.7, 21.0] and −18.53 mmHg, 95% CI [-21.0, −15.7]. The model displayed proportional bias as the slope of the regression of the differences of observed and predicted values by the mean of values was different from 0 (0.36, 95% CI [0.23, 0.49], *p* < 0.001). Observed and predicted AOP values were equivalent, *t* (152) = −9.985, *p* < 0.001, given an equivalence region −7.49 to +7.49 mmHg. The 90% confidence interval for the mean difference between observed and predicted AOP values was −1.25 and +1.25 mmHg, which was well within the selected equivalence region.

#### Internal validation

Distributions for each of the performance measures across internal validation samples for the equation to predict AOP with an 11 cm cuff are shown in Figures 3c–f. The mean model *R* ^*2*^ *adj* was 0.67 ± 0.09, 95% CI [0.66, 0.67], RMSE was 11.7 ± 8.8, 95% CI [11.2, 12.2], CITL was −6.71 ± 25.2, 95% CI [-8.3, −5.1], and calibration slope was 1.03 ± 0.14, 95% CI [1.02, 1.04]. Accordingly, there were small differences between the apparent and optimism adjusted performance measures (*R* ^*2*^ = −0.01, RMSE = −0.02 mmHg, CITL = −2.67, calibration slope = −0.02) indicating minimal overfitting.

For the 13 cm cuff, distributions of the performance metrics across internal validation samples are presented in Figures 4c–f. The mean model *R* ^*2*^ *adj* was 0.64 ± 0.09, 95% CI [0.63, 0.64], RMSE was 8.94 ± 6.25, 95% CI [8.56, 9.33], CITL was 0.83 ± 21.3, 95% CI [-0.49, 2.15], and calibration slope was 1.00 ± 0.13, 95% CI [0.99, 1.01]. Therefore, there were small differences between the apparent and optimism adjusted performance measures (*R* ^*2*^ = 0.00, RMSE = −0.05 mmHg, CITL = −1.72, calibration slope = −0.01) indicating minimal overfitting.

Performance measure distributions across internal validation samples for the 18 cm cuff prediction equation are illustrated in Figures 5c–f. The mean model *R* ^*2*^ *adj* was 0.52 ± 0.13, 95% CI [0.51, 0.53], RMSE was 7.10 ± 5.51 mmHg, 95% CI [6.76, 7.45], CITL was 0.11 ± 25.8, 95% CI [-1.49, 1.72], and calibration slope was 0.99 ± 0.16, 95% CI [0.98, 1.00]. There were small differences between the apparent and optimism adjusted performance measures (*R* ^2^ = −0.01, RMSE = −0.08 mmHg, CITL = −0.99, calibration slope = −0.02) indicating minimal overfitting.

## Discussion

In the current investigation we compared predictors of lower-limb AOP between a variety of commonly used cuff widths (11, 13, 18 cm) for performing exercise with BFR (Part 1). Additionally, we developed and validated prediction equations to estimate lower-limb AOP for these cuffs (Part 2). Our main findings were that 1) the relationship between anthropometric and hemodynamic variables with lower-limb AOP depended on cuff width, 2) TC was the strongest predictor of AOP when using narrower cuffs, but its influence decreased as cuff width increased, with SBP becoming a stronger predictor, and 3) prediction equations containing TC, measures of blood pressure, age, and sex provided acceptable estimates of lower-limb AOP for 11, 13, and 18 cm cuffs that appear to be internally valid. Collectively, these results suggest that methods used to set cuff pressures during exercise with BFR should consider the width of cuff and that our prediction equations provide a valid way to estimate lower-limb AOP for a variety of cuff widths without the need for direct measurement.

### Part 1- predictors of AOP

Our regression models containing the predictor variables of TC, SBP, DBP, age, and sex explained approximately 60%–70% of the total variance in lower-limb AOP across cuff widths ranging from 11 to 18 cm. These results generally support previous findings for 11 cm (de Queiros et al., 2024), 13 cm (Loenneke et al., 2012), and 18 cm cuffs (Cirilo-Sousa et al., 2019; de Queiros et al., 2024; Wedig et al., 2024). Overall, the models explained more variance in AOP with narrower cuffs, explaining 70%, 66%, and 61% of the variance for the 11, 13, and 18 cm cuffs, respectively. Thigh circumference consistently emerged as a significant predictor across all cuff widths, uniquely accounting for 11%–36% of the total variance explained by our models, which supports previous work in 11 cm (de Queiros et al., 2024), 13 cm (Loenneke et al., 2012), and 18 cm cuffs (Cirilo-Sousa et al., 2019; de Queiros et al., 2024; Wedig et al., 2024). Additionally, SBP was also identified as a significant predictor of AOP in all cuff widths and uniquely accounted for 2%–12% of the total variance explained by our models. In contrast, DBP, age, and sex were not consistent predictors and uniquely accounted for relatively small amounts of variance in AOP (<3%). Taken together, our findings across 11, 13, and 18 cm cuffs, along with previous data involving cuff widths from 5 to 18 cm (Cirilo-Sousa et al., 2019; de Queiros et al., 2024; Loenneke et al., 2015; Loenneke et al., 2012; Wedig et al., 2024), suggest that anthropometric and hemodynamic variables are the primary determinants of lower-body AOP, while sociodemographic factors have little unique influence once contributions of limb size and blood pressure are accounted for.

This is the first investigation to directly and comprehensively compare the influence of TC, SBP, DBP, age, and sex on lower-limb AOP in 11, 13, and 18 cm cuffs within a single group of participants. In agreement with previous findings (Crenshaw et al., 1988; de Queiros et al., 2024), our results suggest that the influence of predictor variables on AOP is dependent upon cuff width. While TC was a significant predictor of AOP for all three cuffs investigated, the amount of variance in AOP uniquely explained by TC decreased with increasing cuff width (36%, 26%, 11% for 11, 13, and 18 cm cuffs, respectively), supporting our hypothesis that TC would be a stronger predictor of AOP when using narrower cuffs. A similar relationship was reported by Crenshaw and colleagues (1988) when investigating the influence of TC alone on AOP in 4.5, 8, 12, and 18 cm cuffs, in which TC explained 79%, 67%, 59%, and 24% of the variance, respectively. Comparably, de Queiros and colleagues (2024) reported that adding TC to a regression model already containing SBP explained more additional variance in AOP for an 11 cm cuff compared to an 18 cm cuff, when assessed in the same seated position used in our study. They also found that TC was the strongest predictor for the 11 cm cuff, but not for the 18 cm cuff, based on standardized ß coefficients. These interpretations should be made with caution, as the relative influence of predictors is conditional on the other variables included in the model and does not reflect their unique contributions. To our knowledge, this is the first study to report the unique and shared contributions of TC to the variance in AOP. Results of our mixed linear effects modeling indicated that there was an interaction effect between TC and cuff width, suggesting that the relationship between TC and AOP varied across the different cuff widths (i.e., the slopes of the lines plotting AOP vs. TC were different between cuff widths; see Figure 2a). For the narrower cuffs, each unit of TC was associated with a larger difference in AOP than those in the wider cuffs. Differences in the relationship between TC and AOP across cuff widths is likely explained by the efficiency with which various cuffs transmit pressure to underlying soft tissues. Wider cuffs have been suggested to transmit force more effectively into underlying tissues (Crenshaw et al., 1988), which would in turn reduce the influence of limb size in determining the amount of external cuff pressure that is required to mechanically compress the underlying vasculature and occlude blood flow. As the proportion of variance in AOP explained by TC decreases with increasing cuff width, the total variance accounted for by the model also declines. This likely explains why our regression models including the same predictor variables (TC, SBP, DBP, age, and sex) explained progressively less variance in AOP as cuff width increased (70%, 66%, and 61% for the 11, 13, and 18 cm cuffs, respectively). While TC’s predictive power diminishes with wider cuffs, the contribution of the remaining variables does not increase proportionally, resulting in a lower overall explanatory capacity.

In agreement with de Queiros and colleagues (2024), SBP became a stronger predictor of AOP as cuff width increased, uniquely accounting for 2%, 6%, and 12% of total variance in 11, 13, and 18 cm cuffs, respectively. These results support our hypothesis that blood pressure would be a stronger predictor of AOP when using wider cuffs. Previous reports (de Queiros et al., 2024; Wedig et al., 2024) have identified SBP as the strongest predictor of lower limb AOP in an 18 cm wide cuff based on standardized ß coefficients. While our findings generally support this, our results show that SBP uniquely accounts for only ∼1% more variance than TC in this cuff, suggesting that despite its relatively greater influence compared to narrower cuffs, SBP and TC are similarly important predictors in this context. Arm and leg blood pressures are closely related (Sheppard et al., 2019). Therefore, brachial SBP reflects the pressure within the arteries of the lower-limb which must be overcome by the cuff to compress and occlude their blood flow. Interestingly, results of our mixed-linear modeling indicated that there was no interaction effect of SBP and cuff width, suggesting that the relationship between SBP and AOP was consistent across the three cuff widths (i.e., the slopes of the lines plotting AOP vs. SBP were not different between cuff widths; see Figure 2b). Accordingly, each unit of SBP was associated with similar differences in AOP for all cuff widths. These data indicate that increases in variance explained by SBP with increasing cuff width may be mostly due to reduced dependence on TC as a predictor rather than a shift toward SBP playing a larger physiological role in determining the pressure required to compress and occlude the vasculature. Taken together, these results suggest that most of the variance in AOP that occurs between cuffs of different width is explained by factors related to the transmission of external force from the cuff to the vasculature (Cuff width x TC). After accounting for this variance, the pressure that must be overcome within the artery (SBP) may be a consistent determinant of AOP regardless of cuff width. Ultimately, these data suggest that methods of establishing cuff pressures during exercise with BFR should consider the width of cuff being utilized. Specifically, for narrower cuffs, methods of setting pressure based on TC alone may be suitable, whereas methods for setting pressure in wider cuffs should be based on both TC and SBP.

### Part 2 - Developing and validating prediction equations

We developed three specific prediction equations to estimate lower-limb AOP for the 11, 13, and 18 cm cuffs. Each equation demonstrated good predictive performance, adequate calibration, and minimal evidence of overfitting, which supports their use for setting individualized exercising cuff pressures during exercise with BFR. Unlike previous AOP prediction models (Cirilo-Sousa et al., 2019; Jessee et al., 2016; Loenneke et al., 2015; Loenneke et al., 2012; Sieljacks et al., 2018), we employed LASSO regression for variable selection and coefficient regularization. LASSO is a robust method for creating parsimonious models and reducing overfitting (Steyerberg et al., 2000), aligning with best practices in prediction modeling (Chowdhury and Turin, 2020). Model performance was evaluated using calibration and Band-Altman Limits of Agreement, as recommended for prediction model assessment (Steyerberg and Vergouwe, 2014) and for determining the level of agreement between continuous measures (Zaki et al., 2012). Lastly, each of our models were subjected to internal validation procedures performed via bootstrap resample, which is consistent with recommended practices for the development of clinical prediction models (Moons et al., 2015; Steyerberg, 2008).

The developed prediction equations demonstrated good model fit, calibration, and agreement with direct measurements of AOP taken using Doppler ultrasound. While each model included slightly different predictor combinations, TC and SBP were consistently retained in the final models across all cuff widths. Model fit tended to be highest for narrower cuffs, which accounted for more total variance in AOP and displayed lower RMSE compared to wider cuffs. Similar to Yamada and colleagues (2022), proportional bias was observed in all models, each with a tendency to overestimate lower AOP values and underestimate higher AOP values. This consistent bias across models may reflect either measurement-related error in predictor variables or unmodeled physiological mechanisms that influence AOP estimation across the range of observed values. Despite the proportional bias, group-level comparisons indicated statistical equivalence between predicted and measured values (within 10% of the mean AOP value determined via Doppler ultrasound) with no systematic bias of under or overestimation. Limits of Agreement ranged from ±18.4 to ±28.6 mmHg and narrowed with increasing cuff width, suggesting slightly greater individual-level precision in wider cuffs. Internal validation showed minimal overfitting and good model stability, as performance for each of the equations remained consistent between the development dataset and random bootstrapped samples. Accordingly, our data supports the clinical utility of these equations, as predictions fell within a practically acceptable range for prescribing BFR pressures and their performance appeared to be stable within our data.

Importantly, these equations are intended to estimate AOP for selecting cuff pressures to be used during exercise with BFR. Evidence indicates that exercising with pressures between 40% and 80% of AOP are effective in promoting training adaptations (Patterson et al., 2019) which represents a wide range of effective pressures that can be utilized. Given that some acute responses (e.g., pain, discomfort, blood pressure) and chronic adaptations (e.g., muscular vs. vascular changes) are pressure-dependent (Mouser et al., 2019; Patterson et al., 2019), the ability to estimate AOP precisely may be valuable. However, due to the observed Limits of Agreement, caution is warranted when selecting pressures at the extreme ends of the recommended pressure ranges when utilizing these equations. As such, when using these three prediction equations, we recommend using a more conservative target zone (i.e., ∼45–70% of estimated AOP) to ensure actual exercising pressures remain within a safe and effective window. It is important to note that AOP can fluctuate over time within individuals, likely due in part to acute changes in hemodynamic variables such as blood pressure (Ingram et al., 2017). Our prediction equations may capture some of this variability by including blood pressure as a predictor; however, other transient physiological factors such as vessel size may also influence AOP (Vehrs et al., 2024) and are not accounted for in our models. While we have not directly tested the reliability of the equations across multiple time points with consistent blood pressure and thigh circumferences, we do not anticipate substantial changes in predictive accuracy provided that current values are measured and entered at each use. To account for temporal changes in AOP, it is essential that updated blood pressure and thigh circumference measurements are used each time BFR is implemented.

To the best of our knowledge, these are the first internally validated prediction equations for estimating AOP across several cuff widths commonly utilized to implement exercise with BFR. They may offer a practical solution for practitioners and researchers lacking access to equipment for directly measuring AOP. Since limited equipment access is a frequently cited barrier to BFR use (Cuffe et al., 2022; Scott et al., 2024), these equations may help to enhance accessibility to BFR in variety of settings.

## Limitations

This study has several limitations that should be considered when interpreting the results. First, while internal validation did not suggest that our model was overly optimistic, we did not reach the *a priori* sample size needed to fully minimize optimism and ensure parameter precision in the 11 and 13 cm cuff width models. Second, while our prediction equations demonstrated good internal validity, they were not externally validated. As such, their generalizability to independent samples remains untested and should be confirmed in future work. Third, AOP and predictor variables were not measured under blinded conditions, and data collection was not standardized for time of day. These factors may have introduced measurement variability or bias. Fourth, the type of cuff used (material and inflation mechanism) was not identical between all three cuff widths evaluated making comparison of predictor variables across cuffs less consistent. Also, these cuffs may differ from other commercially available cuffs of the same width in material, bladder type and position, shape and contour, which could influence AOP and limit the generalizability of our equations to other devices. Fifth, although we aimed to increase female representation in our sample, we did not control for menstrual cycle phase, which may influence hemodynamic variables and thus affect the relationship between predictors and AOP. Finally, our models were developed using a relatively homogenous sample of young, healthy adults. We did not collect data on participants’ racial or ethnic backgrounds, which limits our ability to assess how these equations might perform across diverse populations or in clinical cohorts with varying health statuses. Importantly, the validity of our prediction equations is limited to individuals whose blood pressure and thigh circumference fall within the ranges observed in our sample. Therefore, caution is warranted when applying these equations to individuals with hypo- or hypertension, obesity, or other conditions associated with larger limb girth or abnormal limb composition. As these populations may face a higher risk of adverse events during BFR exercise, we do not recommend using our equations in such cases. Instead, more precise or validated methods of direct AOP determination should be used to set cuff pressure. Future research should aim to validate these equations in external, more diverse populations that better reflect those who may benefit most from BFR training and to assess their performance across different cuff types. These efforts are essential to fully establish the clinical utility and broader applicability of our models.

## Summary

For this study, we compared predictors of lower-limb AOP across commonly used BFR cuff widths (11, 13, 18 cm) and developed prediction equations to estimate AOP for each cuff. Our findings demonstrated that anthropometric and hemodynamic variables, particularly TC and SBP, are the primary determinants of AOP, with their relative influence varying by cuff width. Specifically, TC was a stronger predictor with narrower cuffs, while SBP became more influential with wider cuffs, underscoring the need to tailor methods for setting BFR cuff pressure to the width of cuff being used. The resulting prediction equations indicated good calibration, acceptable agreement with direct AOP measurements, and minimal overfitting, supporting their use as practical tools for estimating AOP when direction measurement is unavailable. To promote safety and effectiveness, we recommend applying a conservative target pressure range (e.g., ∼45–70% of estimated AOP) when using these equations. Finally, these results may help to improve accessibility to individualized BFR pressure prescription and facilitate broader implementation of BFR training in rehabilitation and sport training settings.

## Data Availability

The raw data supporting the conclusions of this article will be made available by the authors, without undue reservation.

## Abbreviations

AOP, Arterial Occlusion Pressure; BFR, Blood Flow Restriction; BMI, Body Mass Index; CITL, Calibration-in-the-large; DBP, Diastolic Blood Pressure; LASSO, Least Absolute Shrinkage and Selection Operator; SBP, Systolic Blood Pressure; TC, Thigh Circumference; TOST, Two One-side t-test; VIF, Variance Inflation Factor.
